# Somatostatin Analogs in the Medical Management of Occult Bleeding of the Lower Digestive Tract

**DOI:** 10.1155/2015/702921

**Published:** 2015-03-09

**Authors:** Julie Martin-Grace, Gianluca Tamagno

**Affiliations:** ^1^Department of Medicine, St. Michael's Hospital, Dun Laoghaire, County Dublin, Ireland; ^2^Department of General Internal Medicine, St. Columcille's Hospital, Loughlinstown, County Dublin, Ireland

## Abstract

The management of occult bleeding from the lower digestive tract can be very challenging. In cases of identified angiodysplasia, the first line management options can be limited by a number of clinical or anatomical factors and an alternative approach might be required, including the use of medications. Somatostatin analogs have been considered as an effective and well-tolerated alternative management option in a number of cases where the endoscopic therapeutic approach is not practical or is unsuccessful, in patients with coagulopathies or who need concomitant anticoagulation or antiplatelet treatment. Despite the lack of double-blinded randomized placebo-control trials and despite the limitations of the available evidence in the literature, including small sample size, variable dosing, and administration routes, there is a consistent agreement in the literature that somatostatin analogs are a safe, well-tolerated, and effective therapeutic option for managing refractory or occult bleeding from the lower digestive tract, with particular reference to patients with angiodysplasia, where endoscopic or surgical management is either not feasible or unsuccessful and other pharmacological options are not preferred.

## 1. Introduction

The management of occult bleeding from the lower digestive tract offers a diagnostic and therapeutic conundrum. The origins of both iron deficiency anemia and positive fecal occult blood testing remain unclear in up to 51% of cases, despite radiological and endoscopic investigations [1]. A single focus or multiple foci of angiodysplasia represent approximately 2–8% of all gastrointestinal bleeding; however, this has been reported as accounting for 70–80% of cases of occult gastrointestinal bleeding [2], mostly affecting the lower digestive tract. In cases of identified angiodysplasia, the first line management options including surgery, interventional radiology procedures, and argon plasma coagulation [3, 4] can be limited by patient factors, procedural risks, anatomical factors such as accessibility, localization of the culprit lesion, and the extension or multifocal nature of the lesions [4], necessitating an alternative management option which includes the administration of drugs. Hormonal therapy with estrogen and progesterone has been explored as a therapeutic option but has yielded disappointing results, in addition to reported associated side effects with serious outcomes, including ischemic stroke and deep venous thrombosis [5, 6]. Thalidomide has been used for its antiangiogenic properties and has been found to be effective in a recent open label randomized control trial [7]. However, thalidomide tolerability due to side effects has limited its use in clinical practice [8]. Somatostatin analogs, namely, octreotide and lanreotide, have been considered as an effective and well-tolerated alternative management option in a number of cases where endoscopic management is not practical or is unsuccessful or in difficulty to manage cases involving associated coagulopathies or need for concomitant use of anticoagulation or antiplatelet treatment. Although no striking evidence of the beneficial effects of such medical approach has been published, the relevant number of cases which have been successfully managed with the administration of a somatostatin analog suggests that a careful and comprehensive review of the literature may be worth at this stage.

## 2. Somatostatin and Its Analogs

In the digestive system, somatostatin is secreted from the mucosa of the gastrointestinal tract, pancreatic islet delta cells, and enteric neurons and exerts various actions mostly through an inhibitory physiological effect on the target cells expressing the somatostatin receptors, a family of G protein-couple receptors characterized by five receptor subtypes. There are a number of proposed mechanisms in which somatostatin analogs may contribute and prevent or reduce the bleeding at the level of the digestive tract. These include improved platelet aggregation [9], decreased splanchnic blood flow [10], inhibition of angiogenesis [11], and inhibition of pepsin, gastrin, and acid secretion [12]. It has been postulated that the differing response to treatment might be dependent on different density and expression of the five somatostatin receptor subtypes in the gastrointestinal tract [13].

## 3. Somatostatin Analogs in the Management of Digestive Tract Bleeding

Somatostatin analogs have a well-established role in the management of esophageal varices and peptic ulcer disease [10, 14]. The first reported use of somatostatin analogs in angiodysplasia or occult bleeding was a small case series described in 1993 using subcutaneous octreotide [15]. To date there have been no randomized control trials of somatostatin analogs for the management of angiodysplasia or occult or refractory lower digestive tract bleeding reported in the literature; however, there are a number of case reports, case series, cohort studies, and meta-analyses supporting the use of this class of medications, mostly octreotide, in such clinical setting [13, 16–23]. Moreover, there has also been positive experiences reported with the administration of somatostatin analogs in cases of occult or refractory bleeding associated with coagulopathies or digestive tract bleeding related to the use of anticoagulant or antiplatelet medications [24, 25].

## 4. Review of the Literature

The published studies, case series, and meta-analysis assessing the role of somatostatin analogs in the management of bleeding from the lower digestive tract are summarized in Table 1. Nardone and colleagues described a case series of 17 patients treated for six months with subcutaneous octreotide 100 *μ*g every eight hours for the management of long-standing gastrointestinal bleeding from a number of different causes including bleeding of obscure origin and angiodysplasia, for whom endoscopic or surgical management has either failed or was not possible [16]. The response to treatment was assessed by hemoglobin levels, iron stores, and fecal occult blood before and after treatment. Octreotide was successful in 10 of 17 patients, with an additional 4 patients obtaining some transient benefit. The average hemoglobin increase was statistically significant and moved from 5.5 g/dL to 11.1 g/dL and so was the average decrease in the required blood transfusion units per year, which decreased from 8.8 to 1.5. The first reported case of effective use of long-acting octreotide (OCT-LAR) administered intramuscularly on a monthly basis was described in 2001 [17]. The use of a long-acting octreotide formulation had a significant advantage in terms of burden of treatment and consequently patient compliance. Three patients were trialed on OCT-LAR 20 mg monthly for one year in a bid to overcome compliance issues with subcutaneous administration, which requires multiple daily doses. Response was measured with reference to hospital admissions, acute bleeding episodes, and units of blood transfused, and the patients were followed up monthly for a minimum of one year. None of the patients suffered any bleeding episodes or required admission to hospital in the period of followup, and all experienced a significant rise in their hemoglobin levels. A prospective cohort study by Junquera and colleagues [19] was the first of its kind to attempt to compare octreotide to a placebo control group, matching the results from the cohort study against the placebo arm of a randomized control trial formerly published by his group, looking at efficacy of hormonal therapy in prevention of rebleeding from angiodysplasia [5]. Thirty-two patients received a minimum of one year of treatment with subcutaneous octreotide 50 *μ*g twice daily. The primary endpoint was treatment failure, defined as any episodes of acute bleeding, or chronic bleeding with positive fecal occult blood, iron deficiency anemia, or hematocrit <30% despite iron therapy, compared with the placebo group of the concurrent hormonal trial. It is also worth noting that there were a statistically significantly higher number of patients with coagulopathies or concomitant anticoagulant therapy in the octreotide arm. A 23% treatment failure rate was demonstrated in the octreotide arm, compared with 48% in the placebo group and this result was statistically significant. There was a lower number of bleeding episodes reported in the treatment group; however, this trend did not reach statistical significance. The probability of remaining free of rebleeding episodes at two years after treatment was 68% in the treatment group, compared with 36% in the placebo control. The treatment appeared to be well tolerated, and 9 out of 13 patients reported mild diarrhea, which was self-limiting in most of the cases. A cohort study of 13 patients suggested that a lower dose of intramuscular OCT-LAR 10 mg is an effective therapy in controlling chronic bleeding caused by angiodysplasia where endoscopic or surgical management is not suitable [13]. In this study, 69% of the patients achieved the final endpoint of hemoglobin levels >12 g/dL and are not requiring iron supplementation or blood transfusion one year after completing a 12-month-long course of OCT-LAR treatment. This effect appeared to be sustained in the 7 patients who were followed up for a subsequent year, suggesting somehow a possible ongoing protective effect extending beyond duration of therapy. However, there is not any evidence for confirming such hypothesis. The effect of OCT-LAR treatment appeared to be immediate, with a statistically significant decrease in blood transfusion requirement, iron supplementation, and admissions to hospital during the year of treatment and there were no reported adverse events. A systematic review and meta-analysis published in 2010 looked at three prospective cohort studies [13, 16, 19] accounting altogether for a total of 62 patients treated with octreotide [20]. The analysis highlighted that despite the fact that there are no randomized controlled trials in the use of somatostatin analogs in gastrointestinal bleeding and that the majority of the evidence comes from anecdotal sources, case reports, or small cohort studies, there is consistency among the literature that such class of medications represents an effective and well-tolerated treatment. The clinical response rate of the pooled data from the three cohort studies analyzed was 0.76 (95% confidence interval 0.64–0.85); however, the small sample sizes, the lack of placebo controls, the differing dosing regimens, and the heterogeneous primary endpoints between the studies may weaken this conclusion. Despite the weaknesses in the available literature, the meta-analysis did suggest that a trial of octreotide is an appropriate option in patients who either have failed endoscopic management or are unsuitable for a surgical approach for other reasons. In more recent times, a cohort of 15 patients was evaluated following a minimum of six-month treatment with OCT-LAR 20 mg monthly or lanreotide 90 mg monthly with a view to assessing the effect of decreasing blood transfusion dependence [21]. Only one patient showed a complete lack of response in decreasing their transfusion requirements. The median decrease of the blood transfusion requirement before versus after treatment was 83.3%, with 5 patients requiring no further transfusions. There was a statistically significant decrease in the number of bleeding episodes and a significant increase in the hemoglobin levels. Once again, there were no reported adverse outcomes. Salgueiro and colleagues also looked at efficacy of OCT-LAR in reducing transfusion requirements in angiodysplasia related-gastrointestinal bleeding in a retrospective cohort study of 16 patients [22]. Two dosing regimens were used, namely, 10 mg or 20 mg intramuscularly on a monthly basis. They reported a statistically significant decrease in both blood transfusion requirements before and after treatment (blood units/month 1.84 versus 0.42) and admissions per month (0.21 versus 0). Interestingly, no difference was observed between the group of patients treated with 10 mg and those treated with 20 mg OCT-LAR; however, both of the patients who suffered adverse events (gallstones, splenic infarct) were treated with OCT-LAR 20 mg monthly. However, this was not a statistically significant association and such observation might be determined by the small numbers involved. The authors proposed the use of a low dose of OCT-LAR 10 mg monthly given the lack of increased efficacy at higher dose and the lower cost of this regime, associated with a potentially lower incidence of adverse events. A recent retrospective study of 98 patients attempted to address the potential risk factors for poor response to treatment [23]. To date this study is characterized by both the largest cohort size and the longest duration of followup. Ninety-eight cases, each with a long history of recurrent bleeding prior to treatment, and thereby acting as their own control, were treated with six months of OCT-LAR 20 mg monthly, and mean follow-up time was 78 months. Patients were divided into full responders (stable hemoglobin and no overt bleeding after one 6-month cycle), partial responders (required 3 cycles of therapy to achieve response), and poor responders. There was a statistically significant rise in mean hemoglobin level and a decreased number of bleeding episodes, hospital admissions, and transfusion requirements across all three groups. They also concluded that chronic kidney disease, chronic obstructive pulmonary disease, and ongoing antiplatelet therapy were all independently associated with poor response to therapy. Finally, it is worth mentioning the existence of a small number of case reports mostly involving patients on antiplatelet agents, anticoagulation, or with comorbid coagulopathy [18, 24, 25]. Bowers and colleagues reported two patients with angiodysplasia in the presence of comorbid von Willebrand disease, both rendered transfusion-independent and suitable for outpatient management with the use of subcutaneous octreotide therapy [24]. Blich and colleagues postulated that 10 hospital admissions and 17 units of blood red cells were avoided by managing a patient with diffuse persistent angiodysplasia who required therapeutic anticoagulation for mechanical heart valve with octreotide [25]. To the best of our knowledge, no clinical experience with the newest somatostatin analog pasireotide for the treatment of bleeding from the lower digestive tract has been published to date.

## 5. Conclusion

In conclusion, despite the fact that there has yet to be a double-blinded randomized placebo-control trial to evaluate and despite the limitations of the available evidence in the literature including small sample size, variable dosing and administration routes, and lack of controlled comparison, there appears to be general agreement across the available literature that somatostatin analogs are a safe, well-tolerated, and effective therapeutic option for managing refractory or occult bleeding from the lower digestive tract, with particular reference to angiodysplasia, in patients where endoscopic or surgical management is either not feasible or unsuccessful. Almost all literature reports are based on the administration of octreotide, either in its short-acting subcutaneous formulation or as long-acting intramuscular drug. A recent retrospective study suggested that almost three quarters of patients who have failed or are unsuitable for other management respond to octreotide and more than half of the responders do that in a full manner [23]. This effect is also seen in more challenging cases such as comorbid coagulopathies and patients who require anticoagulation or antiplatelet therapy despite ongoing bleeding. In the clinical setting of lower digestive tract bleeding, somatostatin analogs appear to be well tolerated, and the efficacy of a monthly administration likely decreases treatment burden. The effect of treatment appears to be sustained beyond the duration of therapy. Given the lack of evidence of increased efficacy at higher doses, it is possible that a low-to-medium dose of OCT-LAR (or maybe lanreotide) can represent an appropriate pharmacological management option for refractory, occult, or diffuse bleeding from the lower digestive tract, where endoscopic or surgical management is not possible and other pharmacological options are not preferred.

## Figures and Tables

**Table 1:** Published studies, case series, and meta-analysis assessing the efficacy and safety of somatostatin analogs for the treatment of bleeding from the lower digestive tract.

Study	Design	Number of patients	Treatment	Comparator	Mean followup	Primary outcome	Results	Adverse events
Nardone et al., 1999 [16]	Prospective cohort study	17	Octreotide 100 *μ*g every 8 hours for 6 months	Pretreatment	12 months	Hemoglobin levels, blood transfusions	Effective	Not reported

Orsi et al., 2001 [17]	Case series	3	OCT-LAR 20 mg monthly for 12 months	Pretreatment	16 months	Hemoglobin levels, blood transfusions, bleeding episodes	Effective	None

Junquera et al., 2007 [19]	Prospective cohort study	32	Octreotide 50 *μ*g every 12 hours for 12–24 months	Placebo arm of another study	13 months	Acute or chronic bleeding, fecal occult blood, iron deficiency anemia	Effective	Diarrhea (30%) Abdominal pain (10%) Cholestasis (10%) Hyperglycemia (10%) Hypertriglyceridemia (6.6%) Cerebrovascular accidents (3.3%)

Scaglione et al., 2007 [13]	Prospective cohort study	13	OCT-LAR 10 mg monthly for 12 months	Pretreatment	33 months	Hemoglobin levels, blood transfusions, fecal occult blood	Effective	Mild diarrhea (1/13)

Brown et al., 2010 [20]	Meta-analysis	62	Octreotide or OCT-LAR, various doses	Placebo arm of another study	Various	Clinical response to treatment, blood transfusions	Effective	Not reported

Bon et al., 2012 [21]	Prospective cohort study	15	OCT-LAR 20 mg monthly or lanreotide 90 mg monthly for more than 6 months	Pretreatment	14 months	Hemoglobin levels, blood transfusions, bleeding episodes	Effective	Not reported

Salgueiro et al., 2014 [22]	Retrospective cohort study	16	OCT-LAR 10–20 mg monthly for at least 12 months	Pretreatment	12 months	Blood transfusions, hospital admissions	Effective	Gallstones (1/16 receiving OCT-LAR 20 mg) Splenic infarct (1/16 receiving OCT-LAR 20 mg)

Nardone et al., 2014 [23]	Retrospective cohort study	98	OCT-LAR 20 mg for 6 months	Pretreatment	78 months	Hemoglobin levels, blood transfusion, hospital admissions	Effective	Self-limiting diarrhea Impaired glucose tolerance (1%) Cholestasis (1%)
